# Alfalfa‐Based Dehydrated Silage Pellet as a Source of Nutrients in Laying Hens: I. Effects on Animal Performance and Egg Quality

**DOI:** 10.1002/vms3.70758

**Published:** 2026-01-30

**Authors:** Abbas Hamim, Sylvestre Habimana, Eric Hatungimana, Georges Daube, Bernard Taminiau, Caroline Douny, Jean‐Luc Hornick, Isabelle Dufrasne

**Affiliations:** ^1^ School of Veterinary Medicine University of Rwanda Nyagatare Rwanda; ^2^ Department of Veterinary Management for Animal Resources, Faculty of Veterinary Medicine, FARAH research unit University of Liège Liège Belgium; ^3^ School of Agriculture and Food Sciences University of Rwanda Musanze Rwanda; ^4^ Department of Food Science, Faculty of Veterinary Medicine, Laboratory of Food Microbiology, FARAH research unit University of Liège Liège Belgium; ^5^ Department of Food Sciences, Faculty of Veterinary Medicine, Laboratory of Food Analysis, FARAH research unit University of Liège Liège Belgium; ^6^ Center for Agronomic Technologies Modave Belgium

**Keywords:** alfalfa, animal performance, gut microbiota, organic layer, silage

## Abstract

This experiment aimed to determine the effect of incorporating 10% (w/w) Alfalfa‐based deshydrated Silage Pellets (ABSP) into a commercial control diet on the production performance of Novogen Brown light layers. Twenty‐one‐week‐old hens were divided into two groups (control—C and treatment—T) using a randomized block design, and the experiment lasted for 4 weeks. Feed intake, live weight and egg parameters were measured weekly. Weight gain was slightly lower in the T group, whereas the feed conversion ratio was improved (*p* < 0.05). Laying rate was similar in both groups. Yolk colour intensity was significantly increased (*p* < 0.001), along with an improvement in the yolk's fatty acid profile, showing lower SFA, higher ω‐3 PUFA and an increased ω‐6 to ω‐3 ratio (*p* < 0.001). Gut microbial communities were analysed through 16S rDNA amplicon sequencing. The results indicated that bacterial diversity was significantly lower at the genus level (*p* < 0.01) in the T group. Positive effects were observed on beneficial bacteria abundance, especially *Lactobacillus* spp., and with a reduction in potentially pathogenic bacteria. These findings suggest that ABSP can replace at least 10% of feed in organic layer diet without compromising production parameters and with positive effects on yolk quality and gut microbial communities.

## Graphical abstract

Alfalfa‐based silage pellets (ABSP) showed significant results regarding production performance, yolk colour and ω‐3 (PUFAs), as well as the ratio ω‐6/ω‐3 (*p* < 0.05). The ABSP group also exhibited a higher abundance of beneficial bacteria and a lower abundance of pathogenic bacteria compared to the control group (*p* < 0.05).

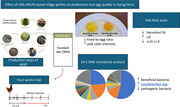

## 1 Introduction

Feed and feeding typically are the largest share of total production costs in organic poultry farming in Western regions, up to 70% according to Van Horne (2020). The situation is further complicated due to the insufficient supply and rising prices of conventional protein sources, such as soybeans and soybean meal, which are mostly imported from outside the European Union (Boerema et al. 2016). Protein dependency raises numerous concerns in terms of poultry farming economics, competitiveness, price fluctuations, environmental impact, transportation and deforestation (Dezat and Gohier‐Austerlitz 2021; Siddiqui et al. 2024; Somagond et al. 2024). The rising global demand for animal products, along with that of conventional feed concentrates, intensifies the competition between humans and livestock for food resources. This underscores the need to identify alternative feed resources that could address this concern. Previous studies have reported that high‐quality forages are potential and sustainable sources of nutrients for organic layer hens farming (Roinsard et al. 2017).

Alfalfa (*Medicago sativa*) forage and by‐products can serve as a viable substitute for conventional feeds. They can help reduce the dependence on imported soybeans as a primary protein source in organic poultry farming, while also lowering production costs (Laudadio et al. 2014; Grela et al. 2020). Additionally, they may improve both egg production and quality (Steenfeldt and Hammershøj 2015; Wüstholz et al. 2017). Alfalfa has been identified as a proportionally richer source of nutrients, such as protein, essential amino acids, minerals, vitamins and dietary fibre, than most unconventional feeds used in poultry nutrition (Blume et al. 2021; Hadidi et al. 2023). Alfalfa is also a rich source of bioactive secondary metabolites (Vahidipour et al. 2024; Khairy et al. 2025). Additionally, supplementing poultry diets with alfalfa meal improved hen health and egg quality, both physically and chemically (Zheng et al. 2019).

To our knowledge, few studies have investigated the potential benefits of using silage‐fermented forage on the performance of organic laying hens. The main objective of the present study was, therefore, to evaluate the effects of partially replacing a conventional diet with alfalfa‐based dehydrated silage pellets (ABSP) as an alternative ingredient and nutrient source on the performance of organic laying hens.

## 2 Materials and Methods

### 2.1 Preparation of the Experimental Feed Ingredients

Alfalfa‐based silage pellets were produced from early vegetative stage (about 4 weeks of regrowth) forage crop harvested at the Center for Agronomic Technologies (CTA, Strée‐Modave, Belgium). The forage mixture consisted of 75% alfalfa, 10% white clover (*Trifolium repens*) and 15% orchard grass (*Dactylis glomerata*). The forage was mowed and pre‐dried in the field. A total of 2 days after harvesting, the forage was baled (one bale) and wrapped with five layers of plastic film. The bale was unwrapped 7 months later. The fodder was then manually sorted to remove fibrous fractions (stalks). It was then dried in a cold‐air dryer until reaching a dry matter content of approximately 80%. The fodder was then pelletized into pellets approximately 2 cm in length and 5 mm in diameter. To maximize microbiota preservation, the pellets were carefully dried at 50°C for 24 h and then cooled to room temperature. The pellets were homogenized, and a representative sample of 100 g was collected for further chemical analysis. Finally, the pellets were ground using a flat‐die grinder.

### 2.2 Experimental Procedure and Data Collection

A total of 40 Novogen Brown Light organic laying hens (Avibel, Zwinjndrecht, Belgium), 17 weeks old, were used in this study. They had been previously vaccinated against *Salmonella enteritidis* and *Salmonella typhimurium*, coccidiosis and avian infectious Bronchitis with Avipro Salmonella duo (Elanco, Antwerpen, Belgium), Paracox‐8 and Nobilis IB GX (MSD, Brussels, Belgium). All vaccines were administered via drinking water.

**FIGURE 1:**
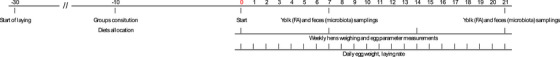
Experimental procedure of the trial.

The experimental procedure used in the study is illustrated in Figure 1. Upon arrival, the hens were group‐housed and acclimated to a commercial organic feed for about 3 weeks. A total of 10 days before the experiment began, the hens were randomly divided into two groups (Control [C] and Treatment [T] and allocated to four blocks per group (five hens each) of similar initial average live weight. The C group was maintained on the standard organic diet, and the T group received a blend consisting of 90% standard diet and 10% (w/w) ABSP. Feed and water were provided on an ad libitum basis, and feed was distributed twice weekly (on Mondays and Thursdays) throughout the entire experiment.

The experimental house consisted of eight boxes (4 m long × 1.25 m wide × 2.8 m high), arranged in two rows of four and separated by a corridor. The boxes were equipped with perches, automated drinkers, non‐automated feeders and nesters. The floor was covered with straw to protect the hen from the cold and other ground‐related issues. All the boxes shared the same air circulation in the building, and the lighting system provided 5–10 lux at hen level with a photoperiod of 16 h of light per day throughout the duration of the trial. The average temperature was 5.8°C during the trial.

The trial lasted for 4 weeks. On Day 0, the hens (21‐week‐old, 1.787 ± 0.122 kg live weight) were weighed, and body weight was recorded individually thereafter at a weekly interval. Eggs were collected, counted and weighed twice daily—in the morning and in the afternoon—using an electronic scale with a precision of 0.01 g. Laying rate was calculated per box as the ratio of the daily egg number to the number of hens. Egg mass production was calculated as the ratio of the total egg weight per box to the number of hens. The feeders were emptied once a week, and the amounts of leftovers were quantified using an electronic scale with a precision of 0.1 g. Weekly feed intake per box was calculated as the difference between the amount of feed distributed and the feed left uneaten. Weekly weight gain and feed‐to‐egg ratio were calculated as secondary parameters.

Once a week, all eggs produced were collected and broken. Haugh Unit (HU) was calculated according to the formula (Silversides 1994): HU = 100 log (*H* − 1.7 × *W* ^0.37^ + 7.6), where *H* = albumen height (mm), *W* = egg weight (g). Shape index was calculated as the ratio of egg width to egg length, multiplied by 100 and yolk index as the ratio of the double yolk height to the sum of the two yolk widths at right angles (Sauter et al. 1951). Yolk colour was estimated using the Roche Yolk Colour Fan with a 16‐scale colour index. The homogenized yolk of each egg used for measurements was sampled, placed in Eppendorf tubes and kept at −18°C for subsequent fatty acid analysis.

On Days 7 and 21 of the trial, three fresh faeces samples were randomly collected and pooled in each box. They were placed in Eppendorf tubes stored at −18°C for subsequent microbiota analysis.

Coccidia count in faecal samples collected from each block was performed weekly. The number of coccidia was counted by using optical microscopy and a Mac Master chamber with a concentrated saline solution (Roepstorff and Nansen 1998).

### 2.3 Ingredient and Chemical Analysis

The dry matter content of ABSP was determined by oven‐drying the sample at 105°C for 8 h. Pellets were analysed according to AOAC methods (AOAC 1990) for crude protein (CP; Method 954.01), ether extract (EE; Method 920.39), ash (Method 942.05) and crude fibre (CF; Method 962.09). The chemical composition of ABSP is presented in Table 1. The chemical composition of the commercial feed was obtained from Société Coopérative Agricole Régionale (SCAR, Herve, Belgium). The ingredient and chemical composition of the diets are shown in Table 2.

**TABLE 1:** Chemical composition of the alfalfa‐based silage pellets.

Components, (% air‐dry basis)	
DM	87.0
CP	24.2
EE	4.9
Ash	14.0
CF	20.0
NDF	52.2
ADF	28.7
Ca	0.80
P	0.49
Na	0.06
Lys^a^	1.00
Met^b^	0.34
NFE^b^	36.9
ME (Kcal/Kg)^c^	2446

Abbreviations: ADF, acid detergent fibre; Ca, calcium; CF, crude fibre; CP, crude protein; DM, dry matter; EE, ether extract; Lys, lysine; ME (kcal/kg), metabolizable energy; Met, methionine; Na, sodium; NDF, neutral detergent fibre; NFE, nitrogen free extract; P, phosphorous.

^a^ Estimated from literature data (Blume et al. 2021).

^b^ NFE = %DM − (%CP + %EE + %CF + %Ash).

^c^ estimated from Carré and Rozo (1990).

**TABLE 2:** Ingredient and chemical composition of the control (C) and treatment (T) diets.

Ingredients (%)	C^a^	T^b^
Wheat	41.0	36.9
Maize	25.0	22.5
Soybean	13.1	11.8
Sunflower cake	10.0	9.0
Mixed grain	2.5	2.3
Organic soybean oil	2.0	1.8
Organic rapeseed meal	1.9	1.7
Salt	0.2	0.18
Additives	0.6	0.54
Calcium carbonate	3.7	3.3
Alfalfa‐based silage pellets	0	10
Total	100	100
Chemical components, (% air‐dry basis)
DM	85.0	85.2
CP	16.9	17.6
EE	5.7	5.6
Ash	7.0	8.3
CF	4.6	5.5
NDF	12.9	16.9
ADF	6.2	8.5
Ca	1.6	0.8
P	0.44	0.44
Na	0.1	0.06
Lys	0.71	0.10
Met	0.30	0.34
NFE^c^	65.8	62.9
ME (kcal/kg)^d^	2850	2810

Abbreviations: ADF, acid detergent fibre; Ca, calcium; CF, crude fibre; CP, crude protein; DM, dry matter; EE, ether extract; Lys, lysine; ME, metabolizable energy; Met, methionine; Na, sodium; NDF, neutral detergent fibre; NFE, nitrogen‐free extract; P, phosphorus.

^a^ From Société Coopérative Agricole Régionale s.c.r.l., Herve, Belgium.

^b^ Obtained by calculation from Footnote a and Table 1.

^c^ NFE = %DM − (%CP + %EE + %CF + %Ash).

^d^ ME (kcal/kg): estimated from Carré and Rozo (1990).

### 2.4 Fatty Acid Analysis

Fatty acid content was analysed by the Folch method (Folch et al. 1957). Two grams of egg yolk were weighed into a 50 mL Falcon tube (Greiner BioOne, Germany), 40 mL chloroform/methanol (2:1) (V/V) was added and the samples were placed on a rotative shaker overnight. Then the samples were filtered using a funnel and a paper filter containing Na_2_SO_4_ into another Falcon tube. Then, 8 mL of a 0.88% KCl solution was added, the mixture was homogenized and it was centrifuged for 10 min at 4000 rpm. Supernatant (methanol and water phase) was discarded using a water pump. Next, 10 mL of the extract was removed from the chloroform phase by placing it in an empty, dried and pre‐weighed glass test tube. The test tube was then placed in an oven at 60°C until all the chloroform had evaporated. The next day, the tube was recovered, and weighed and the fatty acid profile was analysed from the fat content according to the protocol described by Douny et al. (2015).

### 2.5 Faecal Microbiota Community Analysis

#### 2.5.1 DNA Extraction and Purification

Three fresh faeces samples were pooled and stored. Then, the faecal DNA was extracted using the OPERON FDB‐5502 benchtop freeze‐dryer, after freezing and drying the DNA of 24 samples that were sequenced in 16 series plus 8 series; only 17 samples were analysed. The extraction was performed according to the protocol described by Yu and Morrison (2004). In summary, the lysis procedure was performed twice, and supernatants were recovered after cell lysis on 0.25 g of samples using lysis buffer (500 mM NaCl, 50 mM Tris‐HCl, pH 8, 50 mM EDTA, 4% SDS sodium dodecyl sulphate) and zirconia beads (0.1 and 0.5 mm) at 70°C for 15 min and centrifugation at 4°C. Nucleic acids were washed with 70% ethanol and dissolved in 100 µL of Tris‐EDTA buffer (TE, 50 mM Tris, 50 mM EDTA). RNA and protein were removed by incubation with RNase and proteinase K without DNase and using a Qiagen extraction kit (QIAamp DNA Micro Kit 50). Extracted DNA was stored at −20°C.

#### 2.5.2 16S rRNA Gene Library Construction and Sequencing

The V1–V3 region of 16S rDNA of each sample was amplified using (5′‐GAGAGTTTGATYMTGGCTCAG‐3′) and reverse (5′‐ACCGCGGCTGCTGGCAC‐3′) primers with Illumina overhang adapters before purification with the Agencourt AMPure XP beads kit (Beckman Coulter, Pasadena, USA). A second PCR round was performed with Primers 1 and 2 of the Nextera XT index for indexing. Final quantification was performed with the KAPA SYBR FAST qPCR kit (KapaBiosystems, Wilmington, USA) for each sample. Sequence reads processing was used according to the protocol described by (Schloss et al. 2009; Neyrinck et al. 2017) using the respective MOTHUR software package v1.47 that was used for alignment, classification and clustering sequences into operational taxonomic unit (OTU) and the VSEARCH algorithm (Rognes et al. 2016) for detecting and removing chimeric sequences. A clustering distance of 0.03 was used for OTU generation. 16S rDNA reference alignment and taxonomical assignation were based upon the SILVA database (v1.38.1) (Quast et al. 2013). The microbiota structure and composition analysis were obtained through a subsample table with 10.000 sequences/samples.

### 2.6 Statistical Analysis

The box served as an experimental unit, except for body weight measurements, which were performed individually. The statistical analysis was conducted using a general linear model, as follows:

*Y_ij_* = *μ* + *α_i_* + *β_j_* (*α_i_*) + *ε_ij_*.

Repeated measures performed on the same experimental unit were analysed using a mixed model with a Type 1 autoregressive or compound symmetry covariance structure. The model was:

*Y_ij_* = *μ* + *α_i_* + *β_j_* (*α_i_*) + *δ_k_* + (*α *× *δ*)*_ik_* + *ε_ijk_*.

Where *Y_ij_* = the studied variable, *μ* = overall mean, *α_i_* = fixed effect of the *i*th treatment (*i* = 1, 2), *β_j_* (*α_i_*) = random effect of the *j*th replication nested in the *i*th treatment (*j* = 1–4), *δ_k_* = the fixed effect of the *k*th repeated measure, *ε_ij_* = residual error, assumed to be normally distributed.

The indicator of variation was the standard error of the mean (SEM). Least squares means were compared using the Student *t*‐test, followed by Duncan's multiple range test, with a significance level set at *p *< 0.05.

To compare statistical differences in bacterial community abundance between diets, the bacterial community abundances in faeces were summarized by phylum and genus, and differences in relative abundances between groups were compared.

## 3 Results

No animal health issues were detected during the study. The results obtained at the end of the trial showed that coccidia OPG counts were not significantly different between the two groups (138 and 275 OPG fresh dropping, respectively, for the C and T groups, both of which are below the potential cut‐off 10.000 OPG required to observe a reduction in the European Production Index in poultry according to (Haugh et al. 2008).

### 3.1 Animal Performance

**FIGURE 2:**
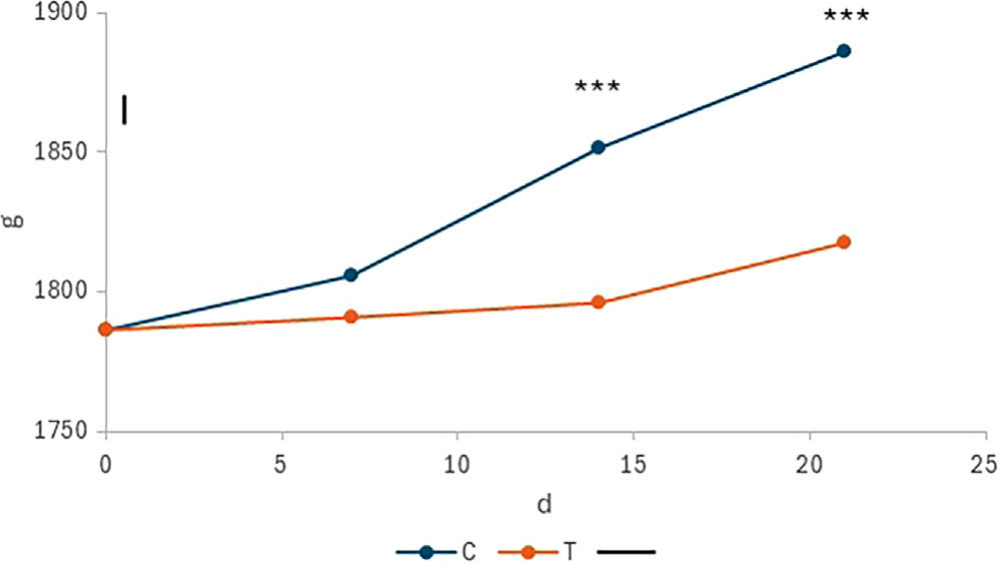
Live weight evolution of the control (C) and the treatment (T) groups. The bar indicates the standard error of the means. The stars indicate within‐time significant differences between groups. Initial hen weights were used as a covariate in the model.

Both groups experienced positive weight gains during the experimental period (Table 3), but the C group exhibited significantly higher weight gain and ADG values than the T group (*p* < 0.001). A significant group × day interaction was observed, the weight difference increasing over time (*p* < 0.01, Figure 2). The egg weight difference between groups was significant but small, favouring C (*p* < 0.03). No egg mass difference was observed. There were notable distinctions between the two groups in terms of feed‐to‐egg ratio, which was lower in the treatment group (*p* < 0.001). The laying rate was numerically higher in the T group, but the difference was not significant (*p* = 0.13).

**TABLE 3:** Laying hens' performance of the control (C) and treatment (T) groups.

Parameter	C	T	SEM	*p* > *F*
Initial weight (g)	1823	1750	28.1	0.06
Final weight (g)	1923	1781	29.2	0.002
Weight gain (g)	99	27	12.6	0.001
ADG (g/day)	4.75	1.48	0.64	0.001
Feed intake (g/day)	147.2	129.6	1.70	0.001
Egg weight (g)	56.4	55.6	0.28	0.03
Egg mass (g)	51.1	51.6	0.64	0.56
Feed‐to‐egg ratio	2.9	2.5	0.06	0.001
Laying rate (%)	90.5	92.9	1.05	0.13

On a DM basis, the leftovers from the experimental group contained significantly more CP (*p *= 0.011) and a trend (*p *= 0.07) for more CF (Table 4). The calculated NFE level tended (*p *= 0.051) to be lower in the leftovers from the experimental group. Overall, they contained more minerals, especially K and Na.

**TABLE 4:** Chemical composition of the leftovers of the control (C) and treatment (T) groups.

Parameter (%)	C	T	SEM	*p* > *F*
DM	85.78	78.75	19.3	0.082
CP	18.23	20.85	4.9	0.011
Ash	9.65	13.95	16.4	0.166
CF	7.55	9.95	7.30	0.073
NFE	65.13	55.20	24.9	0.051
Ca	1.97	2.78	4.3	0.290
P	0.57	0.60	0.18	0.285
K	0.97	1.47	0.83	0.008
Na	0.12	0.20	0.16	0.013
Mg	0.22	0.27	0.13	0.053

Abbreviations: Ca, calcium; CF, crude fibre; CP, crude protein; DM, dry matter; K, kalium; Mg, magnesium; Na, natrium; NFE, nitrogen‐free extract; P, phosphorus.

### 3.2 Physical Characteristics of Eggs

HU, yolk index, shell weight and shell thickness were not significantly different between the two groups (Table 5). The shape index was, however, higher in the C group. Yolk colour was strongly influenced by the treatment, the T group showing a significantly higher value compared to the C group (*p* < 0.001). No group‐by‐measurement × day interaction effect was observed on the parameters.

**TABLE 5:** Physical parameters of a egg of the control (C) and treatment (T) groups.

Parameter	C	T	SEM	*p* > *F*
Yolk colour	7.62	10.4	0.16	0.001
Haugh unit (HU)	103.1	107.4	0.10	0.63
Yolk index	0.522	0.525	0.01	0.58
Shape index	80.4	78.7	0.46	0.011
Shell weight (g)	7.85	7.63	0.12	0.18
Shell thickness (mm)	0.379	0.382	0.0059	0.78

### 3.3 Chemical Characteristics of Eggs

The effect of ABSP on the total fatty acids concentration in egg yolk and their individual and family contribution to fat is shown in Table 6. Total FA represented approximately 35% of yolk, irrespective of group or week. Some minor fatty acids were not consistently detected in the samples. Among the saturated fatty acids, C16:0 was the most represented, followed by C18:0 and their concentration was lower in the T group than in the C group (*p* < 0.05 and 0.001, respectively). Differences decreased over time for C16:0 but increased for C18:0 (*p* < 0.05).

**TABLE 6:** Fatty acid content in yolk (g/100 g yolk lipids) of the control (C) and treatment (T) groups.

	Day		Group		*p* > *F*			
Fatty acid	7	21	C	T	Day	Group	Day × group	SEM
Total (% yolk)	36.0	34.3	35.5	34.8	0.12	0.48	0.34	1.08
Saturated fatty acid (SFA)								
C10:0	nd	0.90	1.01	0.78		0.009		0.055
C14:0	0.36	0.30	0.34	0.31	0.14	0.41		0.030
C16:0	20.3	18.9	20.1	19.0	0.004	0.016	0.024	0.47
C18:0	9.00	8.11	8.75	6.73	0.001	0.001	0.014	0.22
Monounsaturated fatty acid (MUFA)								
C16:1	2.36	2.02	2.16	2.21	0.008	0.684	0.350	0.12
C18:1:9c	41.9	40.4	40.9	41.3	0.021	0.52	0.410	0.62
C18:1:11c	1.93	1.89	1.92	1.90	0.740	0.790		0.051
Polyunsaturated fatty acid (PUFA) ω‐6								
C18:2:9c:1c	20.2	21.1	20.3	21.0	0.180	0.290	0.120	0.69
C18:3	0.30	0.33	nd	0.32				0.025
C20:4	2.42	2.80	2.63	2.59	0.001	0.64	0.140	0.089
Polyunsaturated fatty acid (PUFA) ω‐3								
C18:3	1.66	1.82	1.44	2.04	0.090	0.001	0.051	0.089
C22:5	0.50	0.71	0.61	0.60	0.077	0.820		0.068
C22:6	1.63	2.51	1.86	2.27	0.001	0.001	0.180	0.087
Sums and ratio								
SFA	29.2	27.3	29.3	27.1	0.003	0.001	0.007	0.62
MUFA	45.0	44.0	44.5	44.6	0.170	0.960	0.670	0.70
PUFA	25.8	28.7	26.2	28.3	0.002	0.022	0.095	0.87
ω‐6	22.6	24.0	22.9	23.7	0.062	0.292	0.081	0.70
ω‐3	3.26	4.74	3.41	4.60	0.001	0.001	0.075	0.12
ω‐6/ω‐3	7.43	5.24	7.31	5.36	0.001	0.001	0.151	0.38
PUFA/SFA	0.89	1.07	0.91	1.06	0.001	0.001	0.005	0.044

No significant effect of the diet was detected on MUFA or individual ω6 PUFA. However, the proportion of ω3 PUFA in yolk fat was significantly higher in the T group than in the C group. Several PUFA proportions increased over time, most notably for C18:3 ω3 in the T group (trend, *p* = 0.051). Overall, the positive effect of ABSP on the ω‐3 proportion in yolk fat tended to strengthen over time (*p* < 0.078), allowing a rise from 0.84% on Day 7 to 1.54% on Day 21.

The sum of the PUFA was higher in T compared to C *(p* < 0.05), detrimentally to the sum of the SFA (*p* < 0.001). The total ω3 was significantly increased by 35% in the T group when compared to the C group, while no significant increase was observed in total ω‐6. Consequently, ω6/ω3 ratio decreased by about 30% in the T group (*p* < 0.001). Overall, the ratio PUFA/SFA was in favour of PUFA in the T group and of SFA in the C group.

### 3.4 Gut Microbial Communities

**FIGURE 3:**
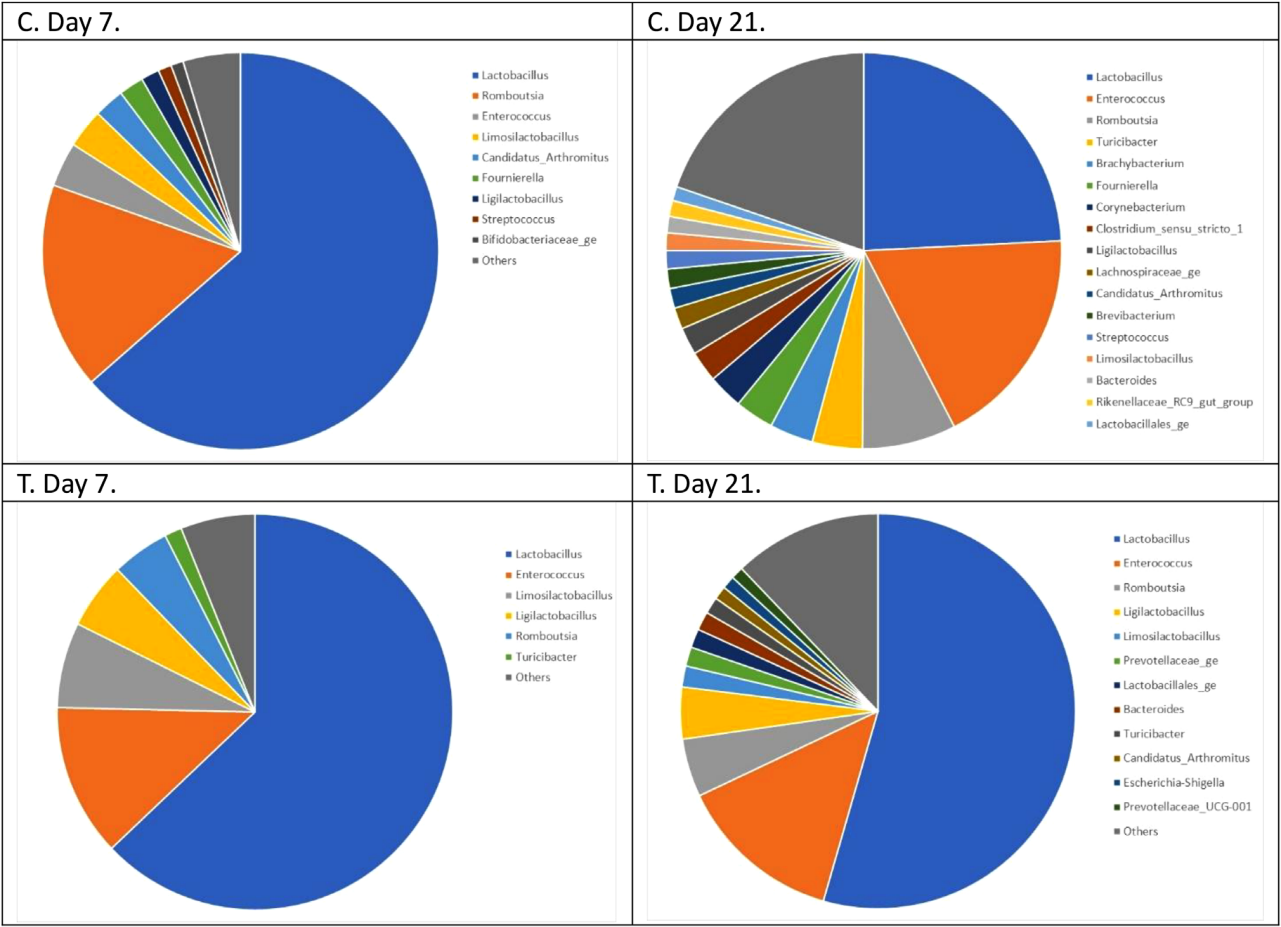
Relative abundance of the main bacterial genera detected in the faeces of the control (C) and treatment (T) groups. The “Others” category included all genera with a relative abundance of less than 1%.

A total of 315 bacterial genera belonging to 14 phyla were detected in the faecal samples. Seven genera were present in all of them. Firmicutes were by far the most represented phylum, followed by Actinobacteriota in the C group and Bacteroidota in the T group (Table 7). *Lactobacillus* was by far the most abundant genus, accounting for 44% of the reads, followed by *Enterococcus* with 14% of the reads (Figure 3). *Romboutsia* represented 5.3% of the reads. *Limosilactobacillus* and *Ligilactobacillus*, two genera from the group of lactic acid bacteria, accounted respectively for 3.7 and 3.1% of the reads.

**TABLE 7:** Phyla and their relative abundance in faeces of the control (C) and the treatment (T) groups. Phyla with less than 50 recovery incidences were discarded.

	C				T			
	Min	Mean	Max	*n*	Min	Mean	Max	*n*
Firmicutes	7260	8828	9944	8	3729	8686	9993	6
Actinobacteriota	48	758	1855	8	1	162	701	9
Bacteroidota	1	302	2353	8	1	931	5527	9
Proteobacteria	4	63	167	8	1	141	398	7
Bacteria_ph	41	41	41	1	1	34	77	9
Patescibacteria	1	6	12	6	2	19	72	6
Cyanobacteria	2	5	14	5	1	15	49	9

*Note*: Sequencing depth achieved: 10,000 reads.

**FIGURE 4:**
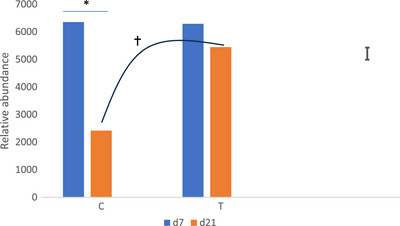
Relative abundance of *Lactobacillus sp*. detected in the faeces of the control (C) and treatment (T) groups, 7 and 21 days after the start of the experiment.

The genera showing significant abundance differences among groups are presented in Table 8. Lower abundances of pathogenic bacteria were detected in the faeces of the T group. In the control group, the relative abundance of *Lactobacillus* decreased significantly from over 50% to less than 25% between Days 7 and 21. In contrast, in the T group, the decrease was not significant, and *Lactobacillus* abundance remained above 50%, tending to be higher (*p* = 0.06) than in the C group (Figure 4).

**TABLE 8:** Bacterial genera and their relative abundance in faeces of the control (C) and the treatment (T) groups.

Genus	C	T	SEM	*p* > *F*
Dietzia	39.1	7.00	2.82	0.01
Facklamia	20.3	2.50	3.97	0.03
Leucobacter	38.3	2.00	7.17	0.01
Microbacterium	29.0	2.75	4.82	0.005
Lactococcus	37.5	2.87	7.33	0.02
Merdibacter	28.9	2.87	4.90	0.005
Microbacteriaceae_ge	21.6	4.37	4.64	0.02

*Note*: Sequencing depth achieved: 10,000 reads.

## 4 Discussion

This study aimed to compare the performance of animals, the physical and chemical characteristics of eggs and the microbiota of laying hens fed either a diet with or without 10% (w/w) alfalfa‐based silage pellets as a source of nutrients.

Weight gain was low in both groups, with a mean daily value averaging less than 5 g/day, but significantly higher in the control group. The difference in live weight between groups stabilized over time, as shown in Figure 2. In modern laying hens, weight increases according to multiphasic Gompertz models and reaches progressively a plateau at around 21–24 weeks of age (Van der Klein et al. 2020). The trial was scheduled at this time to minimize the potential interaction between time and treatment effect.

A slightly lower weight of the hens at the beginning of the laying period may be desirable, as it could reduce the energy requirement for maintenance, thereby improving egg‐to‐feed conversion ratio at a similar laying rate. Lighter Lohmann White hens have been reported to exhibit higher laying performance (Lacin et al. 2008). The inclusion of ABSP in the diet significantly reduced daily feed intake in the treatment group. This may seem surprising given that the two diets contained similar levels of metabolizable energy. One potential explanation is that the experimental diet contained more NDF due to the inclusion of alfalfa. Additionally, the effect of bioactive compounds can vary according to their dose and components (Chen et al. 2023). Alfalfa contains secondary metabolites, such as saponins, that can have an adverse effect on feed intake. He et al. (2021) observed an increase in feed intake in broilers receiving a diet containing 7.5% alfalfa meal compared to a control group. They suggested that insoluble fibre content may be responsible for higher feed intake. Unfortunately, it was not possible to measure the digestibility of the diets in the present experiment. This could be the focus of future investigations.

Egg weight was significantly reduced—at 1.42%—in the T group, while egg mass remained similar. This is explained by the numerically higher laying rate, which is close to a trend in animals that received ABSP. Consequently, feed‐to‐egg conversion was significantly lower in the T group compared with the C group. Improved feed efficiency due to alfalfa supplementation was also observed by Kwiatkowska et al. (2017), who suggested that a variety of bioactive compounds in alfalfa may enhance digestion and the utilization of dietary nutrients. Higher laying performance could be attributed to phytoestrogenic compounds in alfalfa, such as apigenins, luteolins and coumestrol (Seguin et al. 2004; Tucak et al. 2018). These compounds exhibit estrogenic and antiestrogenic effects, potentially increasing the laying rate in hens. There is a limited amount of research on fermented forages and their chemical constituents. Complex interactions between host, microbiota and secondary metabolites of plants have been described in mice (Hu et al. 2023). It cannot be excluded that silage processing adds additional interaction steps at this level. A comprehensive understanding of such complex interactions would require advanced technologies and approaches (see for example Peng et al. 2024).

Hence, the animals receiving ABSP did not experience a decline in performance and appeared to allocate a greater proportion of their diet to egg production rather than growth. Englmaierová et al. (2019) observed a drop in laying rate and an increase in feed‐to‐egg ratio when 4% dehydrated alfalfa was included in the laying hen diet. By contrast, Khajali et al. (2007) reported that 10% alfalfa in the diet did not affect laying, feed intake, or feed conversion ratio.

Other factors may also explain the result of the present study. First, the quality of alfalfa used in our experiment was high because it was harvested at an early stage. Stem removal is another factor that increases the quality of the ingredient. Moreover, forage fermentation during the ensiling process may have contributed to its pre‐digestion, helping the animal tolerate dietary fibres and antinutritional factors (Tian et al. 2022). Additionally, the presence of beneficial bacteria, such as *Lactobacillus* spp., which are part of the microbiota, resulting from forage fermentation, may have contributed to improving animal health. The chicken intestinal microbiota is vital for health, nutrient absorption and immune function (Choi et al. 2015). It has also been reported that the gut microbiota of laying hens plays an essential role in improving nutrient utilization (Dai et al. 2020), production performance (Tian et al. 2022), disease control and immune system regulation (Pan and Yu 2014; Shang et al. 2018), as well as egg quality (Xiao et al. 2022).

Chemical analysis of the leftovers revealed a significant difference between the control and treatment groups. The leftovers in the T group contained significantly more CP, K and Na, less NFE (*p* < 0.01), and a trend for more CF. In general, forages contain high potassium levels (Sulaiman and Al‐Sardary 2021). The ratio of leftovers to feed CP concentration was higher in the T group when compared to group C. This suggests that ABSP was less palatable than the standard diet and probably was retrieved in higher proportions in the leftovers. The results suggest that hens in the T group exhibited a more pronounced feed‐sorting behaviour than those in group C.

A highly significant difference in yolk colour was observed between the two groups, with yolks being noticeably more pigmented in the treatment group than in the control group. This was expected and can be attributed to the presence in ABSP of pigments such as β‐carotene and xanthophyll, which are known to affect yolk colour (Laudadio et al. 2014). Cui et al. (2022) also reported an effect on yolk colour with increasing legume content in laying hen diet. Our results are also in agreement with those reported by Sufe et al. (2023), in hens fed *Moringa oleifera* or Alfalfa leaves.

No other physical parameters differed between groups, except for the shape index, which suggests that eggs from the T group were more compact. The interpretation of this result remains unclear.

The findings showed that ABSP enhanced egg quality by altering the fatty acid composition of the yolk. Since the total fatty acid proportion of the yolk was similar between the two groups, it was not helpful to compare the proportion of fatty acids in the yolk among the groups. In the total yolk fatty acid, the largely represented SFA—C16:0 and C18:0—showed lower values in the T group, particularly for C18:0. No effect was observed on MUFA or on ω‐6 PUFA, but ω‐3 content was strongly increased by 35%, due to 18:3 increase and—to a lower extent—to that of C22:6. Consequently, the ω‐3 increase accounted for 57% of the total PUFA increase. Such observation is surprising given the relatively low level of ABSP incorporation in the diet. However, alfalfa has been reported to contain high levels of ω‐3 fatty acids (Dal Bosco et al. 2014). Another unexpected result is that the ensiling process does not seem to alter Alfalfa's capacity to provide PUFAs to the animal. In the present experiment, the effect of ABSP on the fatty acid profile in egg yolk is highly desirable for dietary human aspects, due to lower SFA, increased sum of PUFA, ω‐3 PUFA and lower ω‐6/ω‐3 ratio. Interestingly, the positive effect of ABSP on the ω‐3 proportion in yolk fat nearly doubled over time. However, an effect on the time was not observed on the ω‐6/ω‐3 ratio as the ω‐6 proportion increased in parallel. Consequently, the PUFA difference between groups increased sharply with time. All the interactions showed at most trends, except for SFAs, which reflected the cumulative effects observed in the other chemical families.

The abundance of the phylum Firmicutes in the poultry intestine is well known (Wei et al. 2013). At a genus level *Lactobacillus spp*. were largely dominant. Silage processing is known to enrich the substrate with *Lactobacillus* species such as *L. plantarum* and *L. buchneri* (Ridwan et al. 2023). The decline in *Lactobacillus* spp. abundance over time in the control group appears to be associated with an increase in alpha diversity during the laying period, reflecting a growing contribution of minor genera. A similar trend was observed in the experimental group, albeit to a lesser extent. Whether the stabilization of microbiota—characterized by a high abundance in Lactobacillus spp., associated with silage pellet feeding—is beneficial for laying hens remains to be determined; however, recent literature appears to support this hypothesis (Gallazzi et al. 2008; Loh et al. 2014; Forte et al. 2016).

The significant differences in the relative abundance of minor bacterial genera were all in the direction of lower values in the T group. *Dietzia* is a potential pathogen in layer hens (Těšický et al. 2024) and was less abundant in the treatment group. *Facklamia* is a genus that is rarely found in poultry, and it has been reported to be pathogenic in humans (Collins et al. 1998; Alonso et al. 2012). *Leucobacter* spp. are mostly found in humans and are still poorly studied in poultry (Boxberger et al. 2023). *Lactococcus* spp. are present in both humans and poultry, with no reported pathogenicity. They can help the intestinal environment resist damage (Boodhoo et al. 2023) and also to improve feed conversion efficiency, feed intake, and growth rate in hens (Stanley et al. 2016). In the present experiment, they were found in lower abundance values in the T group. *Microbacterium* spp. were significantly lower in the T group (*p *< 0.005). Their abundance contributes to the growth of the hens (Zhang et al. 2021).

The lower abundance of certain bacterial genera in the T group is likely a consequence of the higher prevalence of *Lactobacillus* species in this group. These bacteria confer health benefits to the host and improve intestinal health in poultry (Mahfuz et al. 2017; Tellez et al. 2012). Naeem and Bourassa (2025) reported the positive effect of *Lactobacillus* spp. on egg production and quality in laying hens. This effect may result from improved nutrient utilization through enhanced carbohydrate breakdown and fermentation, as well as increased nutrient absorption. A reduction in gastrointestinal disorders due to competition with harmful microbes may also be suggested. Additionally, the abundance of *Lactobacillus* spp. helps to strengthen the intestinal barrier and to modulate the immune response. As a result, it improves hen performance and reduces their susceptibility to the disease. Alaqil et al. (2020) observed that *Lactobacillus* spp. was associated with modulation of humoral and cell‐mediated immune responses and with increased productive performance of laying hens. These findings are consistent with reports that supplementation of diets with *Lactobacillus* bacteria modulates the structure of caecal microbiota, promoting short‐chain fatty acid production, and improving gut health in poultry (Wang et al. 2024). Moreover, the presence of *Lactobacillus* spp. can lead to increased egg production and improved egg quality. Notably, *Lactobacillus* spp. have been shown to significantly improve egg production in laying hens, decrease feed conversion ratio, and enhance eggshell quality (Lim et al. 2024; Liu et al. 2023). The present study has also revealed the presence of Merdibacter and Microbacteriaceae_ge bacteria of unknown genus in poultry. These bacteria have been isolated from the human ileum (Anani et al. 2019) and the environment, respectively. Finally, the potential of silage pellets to exert prebiotic, probiotic, or even postbiotic effects in relation to lactic acid bacteria remains to be determined.

## 5 Conclusion

Feeding ABSP to laying hens has been shown to positively affect production performance and the physico‐chemical properties of eggs of organic hens. The results demonstrate that ABSP can increase yolk content in ω‐3 and improve the ratio ω‐6/ω‐3. Alfalfa silage appeared to stabilize the bacterial communities, favouring *Lactobacillus* species, with a positive impact on microbiota fitness. Further research is essential to assess the optimal utilization of ABSP in formulating layer feed.

## Author Contributions

**Abbas Hamim**: conceptualization, formal analysis, data collection, methodology, software, writing – original draft, writing – review and editing. **Sylvestre Habimana**: reviewing. **Eric Hatungimana**: reviewing. **Georges Daube**: laboratory analysis, reviewing, **Bernard Taminiau**: laboratory analysis, writing – review. **Caroline Douny**: laboratory analysis, writing – review. **Jean‐Luc Hornick**: conceptualization, resources, supervision, validation, methodology, software, writing – original draft; writing – review and editing, **Isabelle Dufrasne**: resources, supervision, administration, validation, methodology, software, visualization, writing – original draft; writing – review and editing.

## Ethics Statement

To conduct this research, the European Union Standards for the protection of animals used for scientific purposes were followed throughout the Ethical Committee of the University of Liège, which authorized the use of the hens in the trials (reference number LA 1610633).

## Conflicts of Interest

The authors declare no conflicts of interest.

## Data Availability

The data of this research are available, and the authors are ready to provide if requested.
